# Effects of Paramylon Extracted from *Euglena gracilis* EOD-1 on Parameters Related to Metabolic Syndrome in Diet-Induced Obese Mice

**DOI:** 10.3390/nu11071674

**Published:** 2019-07-21

**Authors:** Seiichiro Aoe, Chiemi Yamanaka, Kotone Koketsu, Machiko Nishioka, Nobuteru Onaka, Norihisa Nishida, Madoka Takahashi

**Affiliations:** 1 Studies in Human Life Sciences, Graduate School of Studies in Human Culture, Otsuma Women’s University, Chiyoda-ku, Tokyo 102-8357, Japan; 2 The Institute of Human Culture Studies, Otsuma Women’s University Chiyoda-ku, Tokyo 102-8357, Japan; 3 Kobelco Eco-Solutions Co., Ltd., Kobe, Hyogo 651-2241, Japan

**Keywords:** *Euglena gracilis*, paramylon, obesity, abdominal fat, PPARα

## Abstract

Paramylon (PM), a type of β-glucan, functions like dietary fiber, which has been suggested to exert a protective effect against obesity. We evaluated the potential beneficial effects of PM powder on obesity in mice. Male C57BL/6J mice were fed a high-fat diet supplemented with either 2.5 or 5% PM powder, extracted from *Euglena gracilis*, for 74 days. Growth parameters, abdominal fat content, serum biochemical markers, hepatic lipid accumulation and hepatic mRNA expression were measured. Dietary supplementation with PM resulted in decreased food efficiency ratios and abdominal fat accumulation. Dose-dependent decreases were observed in postprandial glucose levels, serum low-density lipoprotein (LDL)-cholesterol, and serum secretary immunoglobulin A (sIgA) concentrations. PM supplementation increased peroxisome proliferator-activated receptor α (PPARα) mRNA expression in the liver which is suggested to induce β-oxidation through activation of acyl-coenzyme A oxidase (ACOX), carnitine palmitoyltransferase (CPT) and fatty acid transport protein 2 (FATP2) mRNA expression. Changes in fatty acid metabolism may improve lipid and glucose metabolism. In conclusion, a preventive effect against obesity was observed in mice given a PM-enriched diet. The mechanism is suggested to involve a reduction in both serum LDL-cholesterol levels and the accumulation of abdominal fat, in addition to an improvement in postprandial glucose concentration.

## 1. Introduction

Metabolic syndrome is a world wide problem; it is a medical term referring to lifestyle related risk factors for arteriosclerosis-related diseases [1]. Obesity and insulin resistance are at the core of most cases of metabolic syndrome; therefore, lifestyle changes, including diet, are strongly recommended. Dietary fiber is one of the best candidates for improving the components of metabolic syndrome [2]. Some dietary fibers reduce the postprandial rise in plasma glucose [3], reduce body weight [4], and improve lipid metabolism [5,6].

Dietary fiber is characterized by water solubility and has different functions. Insoluble dietary fiber has shown favorable results in the treatment of irritable bowel disease [7] and induced a significant increase in fecal bulk, a reduction in intestinal transit time, and a significant increase in the frequency of bowel movements in healthy people [8]. In another study, soluble dietary fiber inhibited carbohydrate and lipid digestion, lipid absorption, reduced the postprandial glucose response, and improved serum lipid levels [9].

Paramylon (PM) is a new source of dietary fiber generated as a characteristic cellular reserve material in *Euglena gracilis*; it is a β-1, 3-glucan arranged in a triple helix and is considered to be an insoluble dietary fiber [10]. Beta-glucan is commonly found in nature, in cereals, fungi and algae, but the structural and physical properties of β-glucans are quite different among the sources. Evidence of any beneficial effects of PM supplementation has not yet been reported. Anti-diabetic effects were not observed in genetically diabetic rats fed a 2% PM diet [11] and recent experiments have shown that PM did not improve obesity or pro-inflammatory status in mice fed a high fat diet [12,13]. The lack of evidence for the beneficial effects of PM is suggested to be caused by insufficient dosage; a recent report suggested that the amount of PM used in the diets of previous studies were insufficient to detect any effectiveness [14]. The dosage of dietary fiber administered via experimental diets is usually 5%, and significant effects have been observed on glucose and lipid metabolism in animal experiments at this dosage [15,16,17]. The cellulose content in the AIN-93 diet is also 5% [18]. Therefore, experiments using adequate amounts of PM in the diet are needed to elucidate the functions of PM as a new dietary fiber.

The purpose of this study was to investigate the effects of 2.5% and 5% PM, extracted from *Euglena gracilis*, in diet-induced obese mice fed a high-fat diet; we measured visceral fat accumulation, cholesterol and glucose metabolism and serum serum secretary immunoglobulin A (sIgA) concentrations. This is the first study to report on the preventive effects of dietary PM against obesity.

## 2. Materials and Methods

### 2.1. Sample Preparation and Chemical Analysis

PM extracted from *Euglena gracilis* EOD-1 was obtained from Kobelco Eco-Solutions Co., Ltd. (Kobe, Japan). The *Euglena gracilis* EOD-1 strain produces high PM yields (70–80%) depending on culture conditions [19]. PM was obtained from cultured *Euglena gracilis* EOD-1 with glucose as the carbon source, under heterotrophic conditions in the dark. *Euglena gracilis* EOD-1 was collected by centrifugation (500× *g*, 4 min) at room temperature and treated with 5 g/L protease (YP-SS; Yakult Pharmaceutical Industry Co., Ltd, Tokyo, Japan) at 50 °C for 2 h before being washed with 1% sodium dodecyl sulfate and distilled water, and then dried. The total dietary fiber (TDF) content of PM was determined to be 97.8% using the method of Prosky et al. [20].

### 2.2. Animals and Study Design

Thirty 4-week-old male C57BL/6J mice were purchased from Charles River Laboratories Japan (Yokohama, Japan) and were maintained on a 12-h light/dark cycle (lights on at 08:00 h). Mice were housed individually in plastic carbonate cages and given free access to water. The animal protocols used were reviewed and approved by the Animal Research Committee of Otsuma Women’s University (Tokyo, Japan) and are in accordance with their animal experimentation regulations. Mice were acclimatized for 7 days before allocation into three experimental dietary groups (*n* = 10): mice were fed either a high-fat diet supplemented with 0% (control), 2.5%, or 5% PM. The control and 2.5% PM diets were supplemented with cellulose to give a total dietary fibre content of 5%. Test diets were prepared according to Table 1. Mice received the experimental diets ad libitum for 74 days. Food intake and body weights were monitored three times a week during the study. Feces were collected over the final 3 days of the study period. The feces were freeze dried after washing the surface with distilled water, milled, and kept at −20 °C until measurement. After fasting for 8 h, mice were sacrificed by isoflurane/CO_2_ anesthesia, then the cecum with digesta, adipose tissue depots: retroperitoneal (including prerenal), mesenteric, epididymal, and liver were dissected and weighed. Small samples of liver tissue (150 mg) were suspended in RNA Stabilization Reagent (RNAlater, Qiagen, Hilden, Germany), and the remainder was freeze-dried, milled, and stored at −20°C until required for cholesterol and triglyceride analysis. Cecum with digesta were stored at −20°C until required for short chain fatty acid analysis. Blood samples, taken from the postcaval vein under isoflurane/CO_2_ anesthesia at sacrifice, were centrifuged, and serum was collected for biochemical analysis.

### 2.3. Biochemical Analysis of the Serum and Concentration of Liver Lipids

Total, low-density lipoprotein (LDL), and high-density lipoprotein (HDL)-cholesterol, triglycerides and free fatty acids were measured in mouse serum using Hitachi 7180 auto-analyzers at the Nagahama Research Institute (Oriental Yeast Co., Ltd., Shiga, Japan). Enzyme-linked immunosorbent assay (ELISA) was used to measure serum insulin (mouse insulin ELISA kit, Shibayagi Co., Ltd., Gunma, Japan) and sIgA concentrations (ELISA Kit for Secretory Immunoglobulin A, Cloud-Clone Corp., Texas, USA). A 2:1 (*v/v*) chloroform-methanol solution was used to extract lipids from the liver [21], which were then dissolved in isopropanol containing 10% polyoxyethylene octylphenyl ether (Triton X-100, FUJIFILM Wako Pure Chemical Corporation, Osaka, Japan). Cholesterol and triglyceride concentration in the lipid extracts were measured enzymatically using the Cholesterol E-test and Triglyceride E-test, respectively (FUJIFILM Wako Pure Chemical Corporation, Osaka, Japan).

### 2.4. Total Fecal Lipid Analysis

Fecal lipids were extracted using a 2:1 (*v/v*) chloroform: methanol mixture under acidic conditions (acetic acid was added to a final concentration of 4%) [22] and determined gravimetrically.

### 2.5. Detection of PM Ultrastructure Recovered from Feces by Scanning Electron Microscopy (SEM)

Intact PM and PM recovered from feces were examined using SEM. Feces were collected and pooled from ten C57BL/6J mice fed a 5% PM diet for 3 days under the same experimental conditions before sacrifice. Lyophilized and powdered feces were treated according to the method of Prosky et al. [20] to collect the dietary fiber. Extracts were washed with distilled water and ethanol, before being dried and coated with osmium. Intact PM was also observed by SEM. Microscopy was performed using a field emission scanning electron microscope (FE-SEM) SU−70–scanning electron microscope (Hitachi High Technologie, Tokyo, Japan) at −1–2 kV accelerating voltage.

### 2.6. Oral Glucose Tolerance Test

Mice were fed the experimental diets for 71 days. After which, they were fasted for 8 h before an oral glucose tolerance test (OGTT) was performed. Blood was collected from the tail tip at 0, 15, 30, 60 and 120 min after oral glucose gavage (1.5 g/kg) and analyzed using a glucose meter (Glutest Ace R, Sanwa Kagaku Kenkyusho Co., Ltd., Aichi, Japan).

### 2.7. Analysis of Short Chain Fatty Acids in Cecal Digesta

The concentration of cecal short chain fatty acids (SCFAs) was determined using a gas chromatography-mass spectrometer system [23]. Ten milligrams of the cecal digesta were homogenized in extraction solution (100 μL of internal standard (100 μM crotonic acid), 50 μL of HCl, and 20 μL of ether) using 5 mm stainless beads (AS ONE Corp., Osaka, Japan). SCFAs in the cecal samples were extracted using a TissueLyser II (Qiagen, Hilden, Germany) at 2000 rpm for 15 min and then centrifuged at 1000× *g* at 25 °C for 10 min. Aliquots (80 μL) of the upper phase (ether layer) were mixed with 16 μL of N-tert-butyldimethylsilyl-N-methyltrifluoroacetamide and heated at 80 °C for 20 min, and then derivatized for 48 h. An aliquot of sample was automatically injected into the inlet of a 7890B GC system (Agilent, Tokyo, Japan) equipped with a 5977A mass selective detector (MSD; Agilent). A DB-5MS capillary column (30 m × 0.53 mm) (Agilent) was used to separate the SCFAs. The oven was programmed to 60 °C for 3 min and increased to 120 °C at the rate of 5 °C/min and then to 300 °C at a rate of 20 °C/min, and finally held at 300 °C for 2 min. Helium was used as a carrier gas at 1.2 mL/min. The temperatures of the front inlet, transfer line, and electron impact ion source were set at 250, 260, and 230 °C, respectively. The ion mass of each SCFA was determined in the selected ion mode. SCFA concentrations were determined by comparing their peak areas with the internal standards, and were expressed as μmol/g of the cecal digesta.

### 2.8. Expression Analysis of mRNAs Related to Lipid and Glucose Metabolism in Liver

Primer sequences are presented in Table 2. Total RNA in the liver was prepared using RNeasy mini kits (Qiagen, Hilden, Germany) according to the manufacturer’s instructions. mRNA expression was measured with an Applied Biosystems Quant3 Real-Time polymerase chain reaction (PCR) System and SYBR^®^ Green PCR Master Mix (Thermo Fisher Scientific, Waltham, MA, USA) using cDNA prepared by real-time (RT)-PCR. The 2^−ΔΔCT^ method was utilized for data analysis where the threshold cycle (CT) indicates the fractional cycle number at which the amount of amplified target reaches a fixed threshold. The ΔCT is the difference in threshold cycles for target genes, using 36B4 as the reference gene. The ΔΔCT is the difference between the ΔCT for treatment diets and the ΔCT for control diet. Relative expression levels are presented as fold changes to the control group (arbitrary unit).

### 2.9. Statistical Analysis

Sample sizes were calculated from our background mice serum data which was based on differences in serum LDL-cholesterol concentrations of 0.06 mmol/L with a standard deviation (SD) of 0.04 mmol/L. The sample size was then calculated with the result that 29 mice were required in total (type I error (α) = 0.05, 1 − β = 0.80): thirty mice (10 mice per group) were used. Data are presented as mean ± standard error of the mean. Dose-dependent effects of paramylon were investigated using a linear regression model. Significant difference (*p* < 0.05) between group means was determined by Williams’s test. The relationships between several parameters related to obesity were evaluated using Spearman’s rank correlation coefficient. JMP (Version 14.0, SAS Institute Inc., Cary, NC, USA) and R software were used to perform the statistical analyses.

## 3. Results

### 3.1. Food Intake, Body Weight and Organ Weight

Food intake, body weight, and food efficiency ratio in mice fed different concentrations of PM are shown in Table 3. There were no significant differences in final weight and body weight gain in the mice fed differing concentrations of PM; however, food intake in the 5% PM group was significantly different when compared with the control group (*p* < 0.05). The food efficiency ratio was dose dependently reduced (*p* < 0.05) in the paramylon groups and a significant difference between the control and the 5% PM group was observed (*p* < 0.05). The organ weights in mice fed paramylon are shown in Table 4. The weights of the cecum with digesta were dose-dependently increased in the PM groups (*p* < 0.01) and the weight was significantly higher in the 5% PM group compared to the control group (*p* < 0.05). Total abdominal, retroperitoneal and mesenteric fat weights were dose dependently reduced in the PM groups (*p* < 0.05) and they were significantly lower in the 5% PM group compared to the control group (*p* < 0.05).

### 3.2. Fecal Total Fat Excretion and Apparent Absorption of Fat

Total fat excretion and apparent absorption of fat into the feces are shown in Table 5. No significant differences were observed in total fat excretion among the groups. The apparent absorption of fat in the 5% PM group was significantly different to the control group, and dose dependency was observed; however, the difference in apparent absorption of fat between the control and the 5% PM group was less than 1%.

### 3.3. Ultrastructure of PM Recovered from Feces by SEM

The ultrastructure of intact PM and PM recovered from feces by SEM are shown in supplementary Figure S1. The recovery of intact PM particles from feces in mice fed the 5% PM diet suggests that PM is resistant to the digestive process and bacterial degradation.

### 3.4. Oral Glucose Tolerance Test (OGTT)

**Figure 1:**
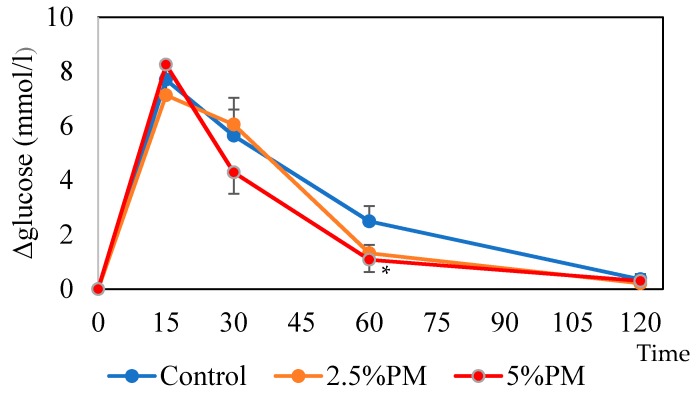
Increase in blood glucose levels in the oral glucose tolerance test (OGTT). Error bars represent standard error (*n* = 10). Paramylon (PM) displayed significant dose-dependent effects in reducing the blood glucose increase level at 60 min (*p* < 0.05). * The increase in blood glucose at 60 min was significantly reduced in the 5% PM group compared with the control group (*p* < 0.05).

The change in blood glucose levels over time was measured with an OGTT (Figure 1). The increase in blood glucose levels after glucose administration was dose dependently reduced at 60 min in the PM groups (*p* < 0.05) and it was significantly reduced in the 5% PM group compared with the control (*p* < 0.05). No significant differences were observed at the other time points or in the incremental area under the curve (IAUC; data not shown) among the groups.

### 3.5. Short-Chain Fatty Acid (SCFA) Concentrations in Cecal Digesta

The concentrations of short-chain fatty acid in cecal digesta are shown in Table 6. Total SCFA concentrations, as well as acetate and *n*-butyrate concentrations, did not differ among the groups. Significant dose-dependent effects of paramylon were observed in increasing propionate concentrations (*p* < 0.05). Propionate concentrations were significantly different in the 2.5% and 5% PM groups compared to the control group.

### 3.6. Biochemical Analysis of the Serum and Liver Lipids

Serum biochemical concentrations are shown in Table 7. PM dose dependently reduced serum LDL-cholesterol levels (*p* < 0.05). In addition, levels were significantly reduced in the 5% PM group compared with the control (*p* < 0.05). There were no significant differences in serum total and HDL-cholesterol, triglyceride, free fatty acid, and insulin concentrations among the experimental groups. Liver lipid levels are shown in Table 8; cholesterol and triglyceride accumulation (mg/liver) and triglyceride concentration (mg/g liver) were not statistically different among the groups.

### 3.7. Expression of mRNAs Related to Liver Lipid Metabolism

**Figure 2:**
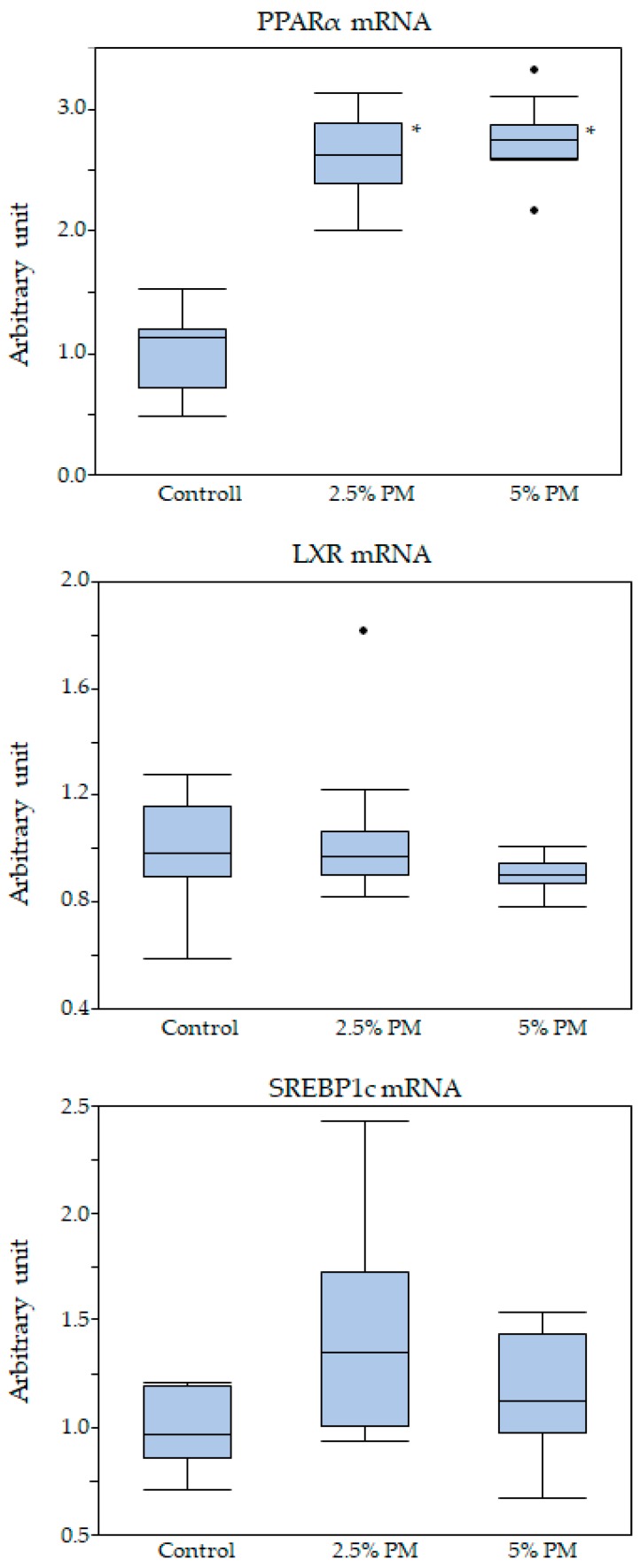
Box-whisker plot of the expression levels of mRNA in liver. * Significant dose dependent up-regulation of PPARα mRNA expression levels was observed in the PM groups (*p* < 0.05).

mRNA expression levels are shown in Figure 2 and Table 9. Significant dose-dependent effects of PM were observed in up-regulating the mRNA expression of peroxisome proliferator-activated receptor α (PPARα; *p* < 0.05): the mRNA expression levels of PPARα were significantly higher in the 2.5% and 5% PM groups compared to the control group (*p* < 0.05; Figure 2). No significant differences in the mRNA expression levels of sterol regulatory element-binding transcription factor 1c (SREBP1c) and liver X receptor (LXR) were observed. PM dose dependently up-regulated the mRNA expression levels of acyl-coenzyme A oxidase (ACOX), carnitine palmitoyltransferase 2 (CPT2) and fatty acid transport protein 2 (FATP2) (*p* < 0.05; Table 9): the mRNA expression levels of ACOX, CPT2, and FATP2 were significantly higher in the 2.5% and 5% PM groups when compared with the control group (*p* < 0.05; Table 9). No significant differences in the mRNA expression levels of carnitine palmitoyltransferase 1 (CPT1), fatty acid translocase (cluster of differentiation36; CD36), lipoprotein lipase (LPL), and fibroblast growth factor 21 (FGF21) were observed. Spearman’s rank correlation coefficients for the relationships between PPARα or serum LDL-cholesterol levels and parameters related to obesity are shown in Table 10. Significant correlations (Spearman’s rank correlation coefficient) between PPARα and each parameter regulated by PPARα, except FGF21, were observed. Significant negative correlations were observed for PPARα vs. LDL-cholesterol levels. Significant positive correlations were observed for abdominal fat weights and liver lipid levels vs. serum LDL-cholesterol levels.

### 3.8. Serum sIgA Concentrations

**Figure 3:**
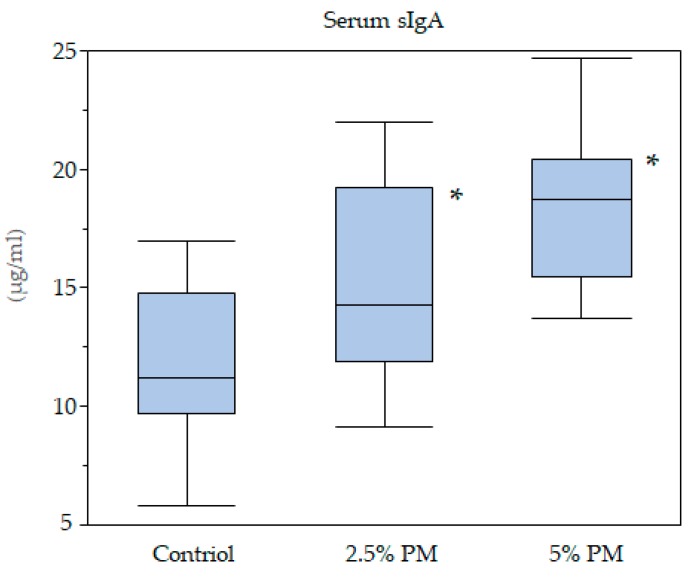
Box-whisker plot of serum sIgA concentrations. * PM increased sIgA levels in a dose dependent manner (*p* < 0.05).

Serum sIgA concentrations are shown in Figure 3. PM dose dependently increased sIgA levels (*p* < 0.05). Serum sIgA concentrations were significantly higher in the 2.5% and 5% PM groups when compared with the control group (*p* < 0.05).

## 4. Discussion

We investigated the effects of PM extracted from *Euglena gracilis* EOD-1 on both lipid and glucose metabolism and abdominal fat accumulation in diet-induced obese mice. This is the first study to report on the preventive effects of dietary PM against obesity. The mechanism involves a reduction in both abdominal fat accumulation and serum LDL-cholesterol concentrations and an improvement in postprandial glucose concentrations. We also determined the characteristics of PM as a dietary fiber source; lipid absorption was hardly influenced by PM intake, and the intestinal fermentability of PM was low. Examination using electron microscopy revealed that PM recovered from feces retained its shape and was resistant to bacterial degradation.

Growth data showed that the reduction in the food efficiency ratio in mice fed PM was dose-dependent; a significant difference was also observed between the control and 5% PM group. It is suggested that reduced lipid accumulation in the abdominal fat organs leads to a reduction in the food efficiency ratio. Significant dose dependent reductions in total, retroperitoneal and mesenteric fat weight were observed in mice fed 5% PM. Dietary fiber has been reported to reduce abdominal fat accumulation through the inhibition of dietary fat absorption [24]. A significant decrease in apparent digestibility of fat was observed with 5% PM supplementation; however, the difference in fat digestibility between the control and 5% PM group was less than 1%. The contribution of PM supplementation to the inhibition of fat absorption may be weak. The lack of reduction in hepatic lipid accumulation is suggested to be partially due to a poor reduction in apparent lipid digestibility.

Sixty minutes after oral glucose administration in mice, the blood glucose level was significantly lower in the 5% PM group compared with the control group. It is suggested that insulin resistance occurs in mice fed PM due to the retardation of gastric emptying [16]. It is also suggested that an increase in colonic fermentation in PM-fed mice may lower the increase in blood glucose due to the increase in SCFAs [12,13]. The production of incretins, such as glucagon-like peptide-1 (GLP-1), promotes insulin secretion and may contribute to improved insulin sensitivity [25]. Significant increases in the concentration of cecal propionate were observed in the 2.5% and 5% PM supplementation groups; however, the contribution of propionate production to the total SCFA concentrations was relatively small. Further studies are needed to clarify the mechanism of improved glucose tolerance by PM intake.

It has been reported that curdlan, produced by *Alcaligenes faecalis* var. *myxogenes*, is also a β-1,3 glucan, but it is composed of linear single chains [26]. Dietary curdlan is reported to be degraded and fermented by intestinal bacteria in the cecum, and may act to improve the intestinal environment via modification in the lower intestine [27]. Therefore, the physiological characteristics of PM and curdlan differ, even though they are both β-1,3 glucans.

Serum sIgA concentrations were dose dependently increased in the PM-supplemented mice, in addition, significant differences were observed between the control group and the 2.5% and 5% PM supplementation groups. The insoluble PM particle itself may exert a direct stimulatory effect on intestinal epithelial cells, which may promote slgA content [28]. PM might promote intestinal immunological activity: *Euglena gracilis* EOD-1 biomass ingestion led to the production of a PM-specific IgA antibody and increased salivary IgA antibody titers in humans [29]. Our data supports these previous reports.

PPARα is involved in many aspects of lipid metabolism [30,31], including fatty acid β-oxidation, synthesis, transport, storage, and lipoprotein metabolism during fasting [32,33]. Activation of PPARα by endogenous ligands or synthetic agonists moderately lowers LDL levels [34]. Serum LDL-cholesterol concentrations were dose dependently reduced in mice supplemented with PM, and significant differences were observed between the 0% group and 5% PM supplementation group. The reduction in serum LDL-cholesterol might be caused by increasing hepatic PPARα expression; this is supported by the significant negative correlation of PPARα mRNA expression vs. LDL-cholesterol levels. Significant positive correlations between retroperitoneal and mesenteric fat weights vs. LDL-cholesterol levels were observed, suggesting that dose dependent reductions in serum LDL-cholesterol levels might cause a reduction in abdominal fat weights. However, we measured only PPARα mRNA expression; qualitative analysis of protein levels will be needed to help elucidate the mechanism of PM’s activation of PPARα mRNA expression.

Liver triacylglycerol production and secretion into VLDL are mainly determined by the fatty acid synthesis rate, which is controlled to a large extent at the level of transcription by both PPARα [35], which stimulates fatty acid β-oxidation, and SREBP-1c [36], which controls fatty acid synthesis. Our results indicated that activation of PPARα might decrease triglyceride accumulation in the abdominal fat tissues by reducing secretion into VLDL, whereas SREBP-1c may not be involved in fatty acid synthesis activation after PM supplementation. Previous reports showed that PPARα regulates the expression of fibroblast growth factor 21 (FGF21) during starvation [37,38]. FGF21 acts as an endocrine hormone targeting various functions, including glucose and lipid metabolism [39]. Our results indicated that mRNA expression of hepatic FGF21 in the 2.5 and 5% PM groups increased by 1.5 times compared to the control group; however, the differences were not statistically significant. Further analyses of serum FGF21 concentrations are needed to confirm the relationship between PPARα mRNA expression and serum FGF21 secretion. It has been reported that PPAR activators inhibit the activation of inflammatory response genes by interfering with nuclear factor-κB (NF-κB) and transcription factor activator protein 1 (AP1) signaling pathways, thereby promoting insulin sensitivity [40]. Improvements in insulin sensitivity caused by PM supplementation might activate PPARα by the same mechanism. Although PPARα is suggested to be activated by high levels of fatty acids present in the fasted liver [41], the exact nature of the endogenous activation signal remains unknown [42]. Further research is needed to elucidate the endogenous ligands promoted by direct PM stimulation of the epithelial cell.

## 5. Conclusions

Dietary fiber has been suggested to exert a protective effect against obesity. PM, from *Euglena gracilis*, is a dietary fibre; however, there has been very little research on its effects. We investigated the effects of 2.5 or 5% PM in diet-induced obese mice fed a high-fat diet; we observed a preventive effect against obesity, which may involve a reduction in both abdominal fat accumulation and serum LDL-cholesterol concentrations and an improvement in postprandial glucose concentrations. Hepatic induction of PPARα mRNA expression is suggested to increase mRNA expression of ACOX, CPT2, FATP2 and induce β-oxidation. Subsequent changes to fatty acid metabolism might cause a series of improvements in lipid and glucose metabolism.

## Figures and Tables

**Table 1:** Composition of the experimental diets (g/kg diet).

	Control	2.5% PM	5% PM
Casein	200	200	200
l-cystine	3	3	3
Corn starch	197.486	196.886	196.286
Dextrinized corn starch	132	132	132
Sucrose	100	100	100
Soybean oil	70	70	70
Lard	200	200	200
Cellulose	50	25	-
PM	-	25.6	51.2
AIN-93G mineral mixture	35	35	35
AIN-93 vitamin mixture	10	10	10
Choline bitartrate	2.5	2.5	2.5
*t-*Butylhydroquinone	0.014	0.014	0.014

PM: Paramylon, TDF 97.7%.

**Table 2:** Primers used in the real-time reverse transcription polymerase chain reaction.

	Forward	Reverse
PPARα	5′-AGGAAGCCGTTCTGTGACAT-3′	5′-AATCCCCTCCTGCAACTTCT-3′
LXR	5′-CCTTCCTCAAGGACTTCAGTTACAA	5′-CATGGCTCTGGAGAACTCAAAGAT-3′
SREBP1c	5′-GGCACTAAGTGCCCTCAACCT-3′	5′-GCCACATAGATCTCTGCCAGTGT-3′
ACOX	5′-CAGCGTTACGAGGTGGCTGTTA-3′	5′-TGCCCAAGTGAAGGTCCAAAG-3′
CPT1	5′-GATGGAGAGGATGTTCAACACTACAC-3′	5′-AGCCCTCATAGAGCCAGACCTT-3′
CPT2	5′-ATCGTACCCACCATGCACTA-3′	5′-TGGCTGTCATTCAAGAGAGG-3′
CD36 (FAT)	5′-TCCCTCTCTGGAGTTCTTGG-3′	5′-TTGCAGCTGAGCAGAAAGAG-3′
FATP2	5′-TTTCCGGTGGAAAGGAGA-3′	5′-AGGTACTCCGCGATGTGTTG-3′
LPL	5′-AGGGCTCTGCCTGAGTTGTA-3′	5′-AGAAATCTCGAAGGCCTGGT-3′
FGF21	5′-CAAGACACTGAAGCCCACCT-3′	5′-TGCCAGGACGCGCTTGT-3′
Reference		
36B4	5′-GGCCCTGCACTCTCGCTTTC-3′	5′-TGCCAGGACGCGCTTGT-3′

PPARα, peroxisome proliferator-activated receptor α; LXR, liver X receptor; SREBP1c, sterol regulatory element-binding transcription factor 1c; ACOX, acyl-coenzyme A oxidase; CPT1, CPT2, carnitine palmitoyltransferase 1 and 2; CD36 (FAT), fatty acid translocase; FATP2, fatty acid transport protein 2; LPL, lipoprotein lipase; FGF21, fibroblast growth factor 21; 36B4, acidic ribosomal protein.

**Table 3:** Body weight gain, food intake, and food efficiency ratio.

	Control	2.5% PM	5% PM	*p* for Trend
Initial weight (g)	19.5 ± 0.2	19.5 ± 0.2	19.5 ± 0.2	n.s.
Final weight (g)	38.3 ± 1.1	39.4 ± 1.0	38.2 ± 0.9	n.s.
Body weight gain (g/day)	0.25 ± 0.01	0.27 ± 0.01	0.25 ± 0.01	n.s.
Food intake (g/day)	2.8 ± 0.1	3.1 ± 0.2	3.1 ± 0.1 *	n.s.
Food efficiency ratio (%)	9.03 ± 0.38	8.83 ± 0.56	8.14 ± 0.24 *	*p* < 0.05

Values are means ± standard error of the mean (SE), *n* = 10. * Significantly different from the control group (Williams’s method; *p* < 0.05), n.s.: not significant.

**Table 4:** Weight of organs.

	Control	2.5% PM	5% PM	*p* for Trend
Liver (g)	1.21 ± 0.04	1.23 ± 0.05	1.19 ± 0.04	n.s.
Cecum with digesta (g)	0.24 ± 0.02	0.28 ± 0.01	0.33 ± 0.02 *	*p* < 0.01
Total abdominal fat	4.13 ± 0.08	3.92 ± 0.15	3.57 ± 0.21 *	*p* < 0.05
Retroperitoneal fat(g)	0.87 ± 0.02	0.81 ± 0.05	0.73 ± 0.04 *	*p* < 0.05
Epididymal fat(g)	2.38 ± 0.05	2.29 ± 0.08	2.21 ± 0.12	n.s.
Mesenteric fat(g)	0.88 ± 0.05	0.83 ± 0.07	0.63 ± 0.07 *	*p* < 0.05

Values are means ± SE, *n* = 10. * Significantly different from the control group (Williams’s method; *p* < 0.05), n.s.: not significant.

**Table 5:** Fecal fat excretion and apparent digestibility of fat.

	Control	2.5% PM	5% PM	*p* for Trend
Fat intake (mg/day)	711.0 ± 32.4	775.8 ± 19.9	747.9 ± 32.1	n.s.
Fecal fat excretion (mg/day)	18.6 ± 2.4	18.9 ± 2.1	25.3 ± 2.1	n.s.
Apparent digestibility of fat (%)	97.4 ± 0.3	97.6 ± 0.3	96.6 ± 0.2 *	*p* < 0.05

Values are means ± SE, *n* = 10. * Significantly different from the control group (Williams’s method; *p* < 0.05), n.s.: not significant.

**Table 6:** Concentration of short-chain fatty acids (SCFA) in the cecal digesta.

SCFA (μmol/g cecum)	Control	2.5% PM	5% PM	*p* for Trend
Acetate	11.9 ± 0.5	12.2 ± 0.5	11.9 ± 0.4	n.s.
Propionate	4.0 ± 0.2	5.0 ± 0.2 *	5.0 ± 0.2 *	*p* < 0.05
*n*-Butyrate	3.8 ± 0.4	4.4 ± 0.4	4.0 ± 0.3	n.s.
Other SCFAs *	1.9 ± 0.1	2.3 ± 0.1	2.5 ± 0.1	n.s.
Total SCFAs	22.3 ± 1.0	24.2 ± 0.9	24.8 ± 0.9	n.s.

Values are means ± SE, *n* = 10. * Other SCFAs, the sum of the concentrations of formate, iso-butyrate, iso-varerate, and varerate is shown. * Significantly different from the control group (Williams’s method; *p* < 0.05), n.s.: not significant.

**Table 7:** Biochemical analysis of the serum.

	Control	2.5% PM	5% PM	*p* for Trend
Total cholesterol (mmol/L)	4.36 ± 0.12	4.56 ± 0.12	4.24 ± 0.13	n.s.
LDL-cholesterol (mmol/L)	0.21 ± 0.01	0.19 ± 0.01	0.17 ± 0.01 *	*p* < 0.05
HDL-cholesterol (mmol/L)	2.21 ± 0.04	2.30 ± 0.03	2.19 ± 0.04	n.s.
Triglyceride (mmol/L)	0.56 ± 0.02	0.52 ± 0.04	0.58 ± 0.07	n.s.
NEFA (μmol/L)	521.6 ± 28.2	477.0 ± 15.8	500.1 ± 11.7	n.s.
Insulin (ng/mL)	1.96 ± 0.32	1.80 ± 0.34	1.34 ± 0.18	n.s.

Values are means ± SE, *n* = 10. * Significantly different from the control group (Williams’s method; *p* < 0.05), n.s.: not significant.

**Table 8:** Liver lipid levels.

	Control	2.5% PM	5% PM	*p* for Trend
Cholesterol (μmol/liver)	2.12 ± 0.10	2.07 ± 0.11	2.03 ± 0.08	n.s.
(μmol/g liver)	1.76 ± 0.07	1.68 ± 0.03	1.72 ± 0.03	n.s.
Triglyceride (μmol/liver)	8.56 ± 1.15	8.72 ± 1.41	7.29 ± 0.96	n.s.
(μmol/g liver)	6.90 ± 0.78	6.82 ± 0.81	6.00 ± 0.59	n.s.

Values are means ± SE, *n* = 10.

**Table 9:** Expression of mRNAs under the control of PPARα in liver.

	Control	2.5% PM	5% PM	Arbitrary Unit (/36B4)
				*p* for Trend
ACOX	1.0 ± 0.2	2.1 ± 0.1 *	2.0 ± 0.1 *	*p* < 0.05
CPT1	1.0 ± 0.2	0.6 ± 0.1	0.5 ± 0.1	n.s.
CPT2	1.0 ± 0.1	1.3 ± 0.1 *	1.3 ± 0.1 *	*p* < 0.05
CD36	1.0 ± 0.2	1.3 ± 0.2	1.4 ± 0.2	n.s.
FATP2	1.0 ± 0.1	2.3 ± 0.2 *	2.0 ± 0.4 *	*p* < 0.05
LPL	1.0 ± 0.2	1.4 ± 0.2	1.3 ± 0.1	n.s.
FGF21	1.0 ± 0.2	1.5 ± 0.3	1.5 ± 0.3	n.s.

Values are means ± SE, *n* = 10. * Significantly different from the control group (Williams’s method; *p* < 0.05). n.s.: not significant.

**Table 10:** Spearman’s rank correlation coefficients for the relationship between PPARα or LDL-cholesterol levels and parameters related to obesity.

	**vs. PPARα mRNA**
ACOX mRNA	0.68 *
CPT1 mRNA	0.55 *
CPT2 mRNA	0.38 *
FATP2 mRNA	0.64 *
CD36 mRNA	0.38 *
LPL mRNA	0.39 *
FGF21 mRNA	0.18
Serum LDL-cholesterol levels	−0.43 *
Abdominal fat weight	−0.16
Blood glucose at 60 min	−0.12
	**vs. LDL-cholesterol**
Retroperitoneal fat weight	0.58 *
Mesenteric fat weight	0.53 *
Liver cholesterol levels	0.59 *
Liver triglyceride levels	0.46 *

* *p* < 0.05 for Spearman’s rank correlation coefficient.

## Supplementary Materials

The following are available online at https://www.mdpi.com/2072-6643/11/7/1674/s1. Figure S1: Intact PM and PM recovered from feces by scanning electron microscopy.
