# Supplementary material for: Effects of Paramylon Extracted from Euglena gracilis EOD-1 on Parameters Related to Metabolic Syndrome in Diet-Induced Obese Mice
Source: Nutrients. 2019 Jul 21;11(7):1674. doi: 10.3390/nu11071674 (PMC6682983; doi:10.3390/nu11071674)
Supplement: Supplementary file 1 [file nutrients-11-01674-s001.pdf]

（A） （B）

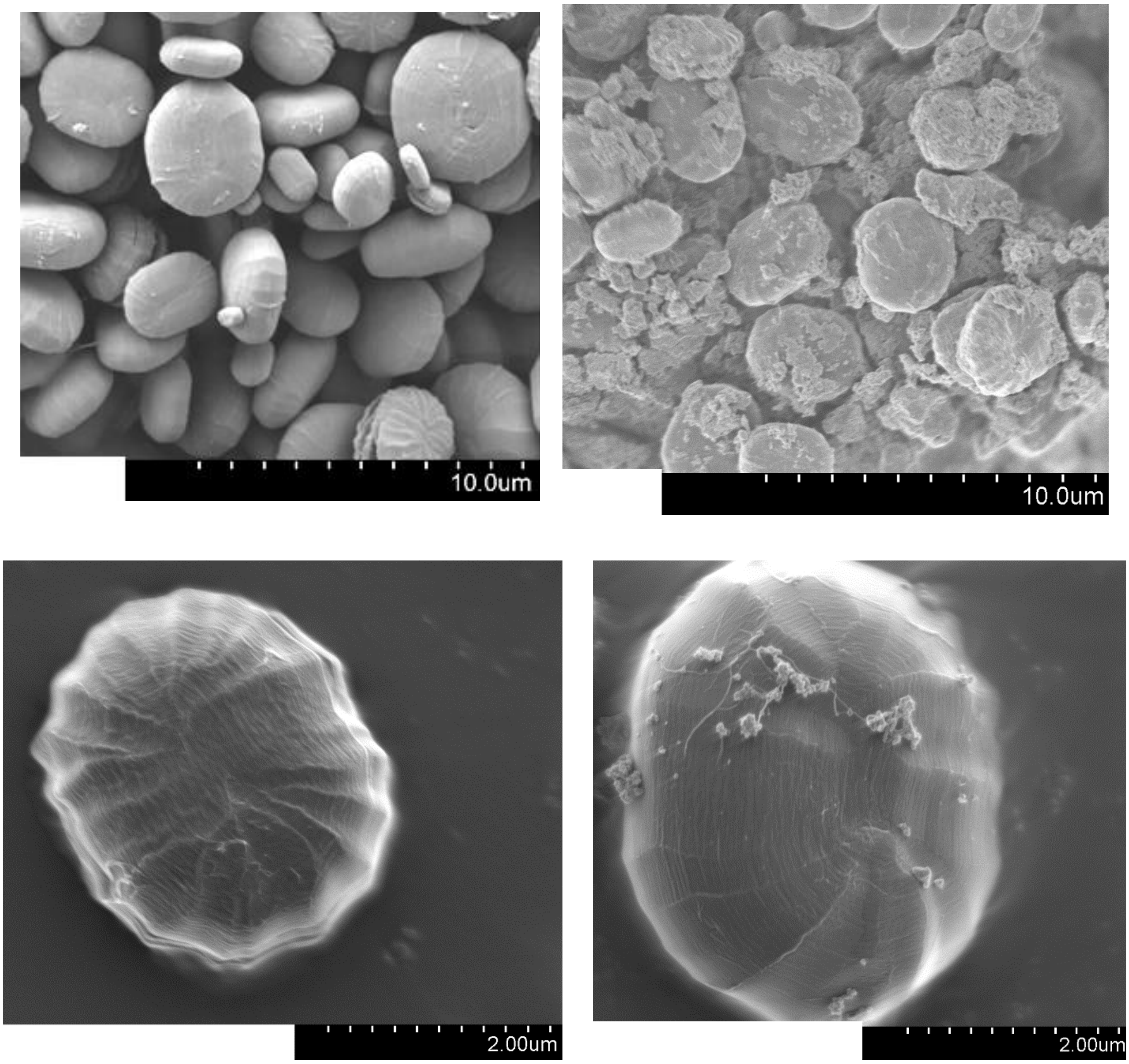

Supplementary Figure S1 Intact PM (A) and PM recovered from feces (B) by scanning electron microscopy.
